# Disseminated Pityriasis Versicolor Associated With Type 2 Diabetes Mellitus: A Clinical Case With Immunometabolic Insights

**DOI:** 10.7759/cureus.95377

**Published:** 2025-10-25

**Authors:** Jesús Iván Martínez-Ortega, Ilse Fernández-Reyna, Alejandra Nicole Macias Quiroga

**Affiliations:** 1 Histology, Autonomous University of Nuevo Leon, Monterrey, MEX; 2 Dermatology, Dermatology Institute of Jalisco, Zapopan, MEX; 3 Mycology, Centro Dermatológico de Yucatan, Mérida, MEX; 4 Dermatology, Hospital Obrero No. 1, La Paz, BOL

**Keywords:** acanthosis nigricans, azelaic acid, diabetes mellitus type 2, insulin resistance, interleukin-13, interleukin-17, itraconazole, malassezia, pityriasis versicolor, ppar gamma

## Abstract

Pityriasis versicolor (PV) is a common superficial fungal infection caused by *Malassezia* species, typically confined to seborrheic areas. Disseminated forms are uncommon and may reflect underlying host or environmental factors. We describe a 40-year-old male construction worker from a tropical region presenting with widespread hypopigmented macules and acanthosis nigricans. Direct microscopy confirmed *Malassezia* infection, and laboratory evaluation revealed previously unrecognized type 2 diabetes mellitus (T2DM) (acylated hemoglobin (HbA1c) 8%, Homeostasis Model Assessment of Insulin Resistance (HOMA-IR) 5.2; insulin resistance threshold > 2.5). Baseline liver function tests were normal. The patient achieved complete resolution after two weeks of oral itraconazole (100 mg twice daily) with concurrent initiation of metformin and dietary management.

This single, hypothesis-generating case suggests that metabolic dysregulation may serve as a contextual rather than causal factor in PV dissemination, acting together with environmental conditions such as heat and humidity that favor fungal proliferation. We propose a speculative “partial-containment” model in which *Malassezia* overgrows within lipid-rich, low-inflammation environments, producing azelaic acid that suppresses melanogenesis and results in hypopigmentation. While causality cannot be determined from a single case, multidisciplinary care and metabolic screening may benefit patients with atypical or extensive PV, and prospective studies are warranted to validate these mechanisms.

## Introduction

Pityriasis versicolor (PV) is a common superficial mycosis caused by *Malassezia *species, commensals of normal skin. Prevalence varies with climate, affecting roughly 1-4% of individuals in temperate regions and up to 50% in tropical, humid settings. Clinically, PV presents as hypo- or hyperpigmented, finely scaling macules on seborrheic sites. In hypopigmented variants, fungal lipases generate dicarboxylic acids (e.g., azelaic acid) that inhibit dopa-tyrosinase and reduce melanogenesis, accounting for pale coloration with typically minimal inflammation.

Generalized or disseminated involvement is uncommon, and whether underlying metabolic conditions or immunosuppression contribute to disseminated presentations remains an area of active investigation [1]. In the absence of a consensus definition, disseminated PV can be operationally described as the involvement of ≥3 non-contiguous body regions beyond typical seborrheic sites, consistent with prior literature on recurrent and disseminated forms [2]. We report a disseminated PV case in an adult and discuss the debated relationship with type 2 diabetes mellitus (T2DM), alongside an immune-metabolic working hypothesis for dissemination.

## Case presentation

**Figure 1:**
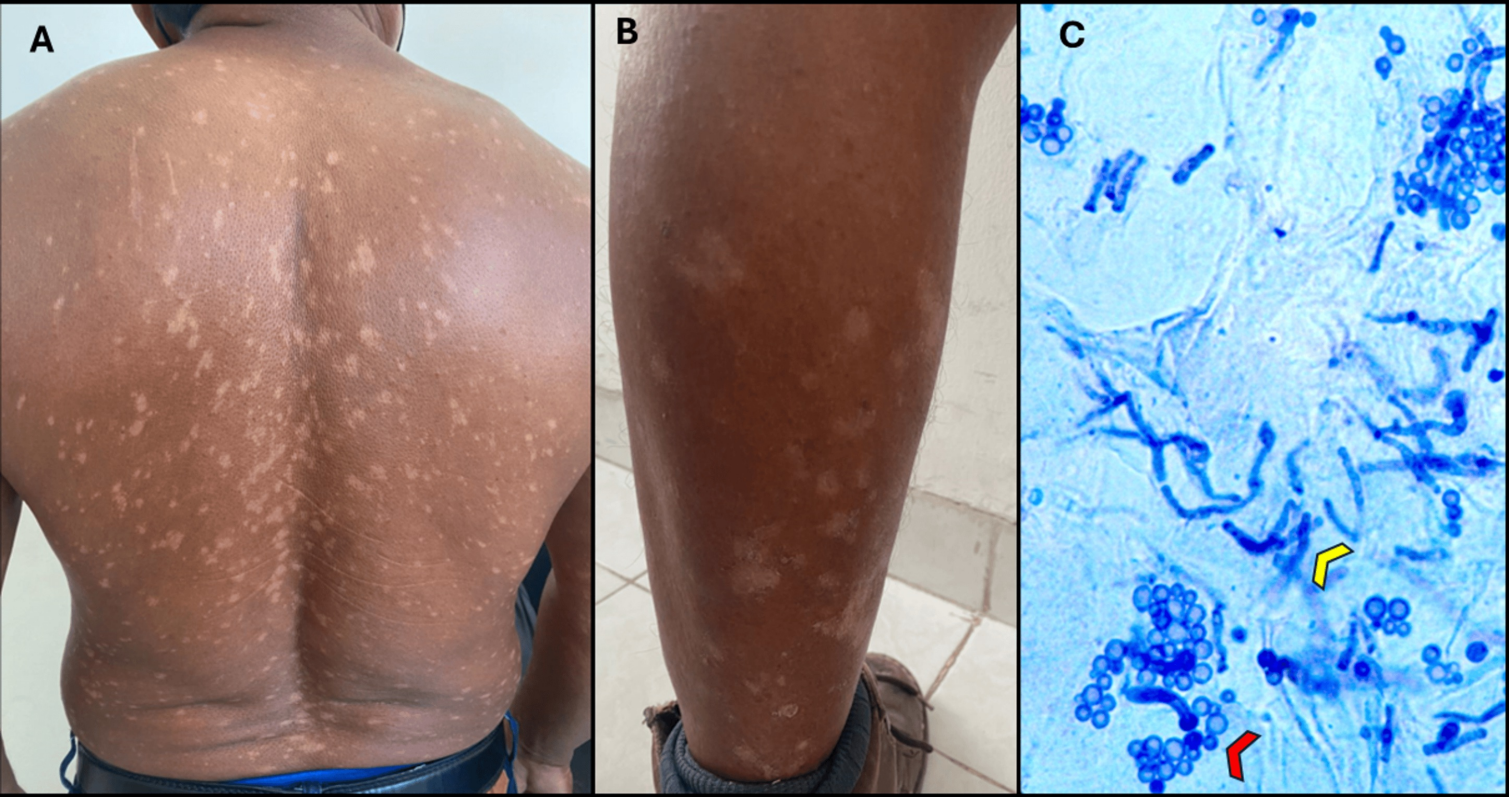
Disseminated hypochromic pityriasis versicolor (A) Multiple hypopigmented macules and patches with fine scaling on the back. (B) Hypopigmented lesions extending to the distal lower extremities, illustrating the widespread distribution from the trunk to all four limbs. (C) Direct microscopic examination of lesional scale with methylene blue stain (1%) at 10× magnification, showing clusters of round spores (red arrowhead) and short hyphae (yellow arrowhead) consistent with *Malassezia *spp. Written informed consent was obtained from the patient for publication of this image.

A 40-year-old male was evaluated at an outpatient dermatology clinic in Mérida, Yucatán, Mexico, a region with a hot, humid climate. He works as a construction laborer, with daily heat exposure, occlusive clothing, and frequent sweating. He presented with a five-month history of hypopigmented spots on the body. Physical examination revealed multiple rounded, finely scaling hypopigmented macules affecting the posterior trunk (Figure 1A), with mild extension to the anterior torso and both upper and lower extremities (Figure 1B). His body mass index (BMI) was 31 kg/m², consistent with obesity. Family history was non-contributory, and the patient denied use of corticosteroids or other medications. Differential diagnoses at this point included vitiligo, progressive macular hypomelanosis, post-inflammatory hypopigmentation, and PV. Additionally, hyperpigmented, velvety plaques with a reticulated pattern were noted on the posterior neck, suggestive of acanthosis nigricans. Direct microscopic examination with methylene blue stain showed clusters of round spores and short hyphae (Figure 1C), confirming *Malassezia *spp. infection.

Laboratory workup is summarized in Table 1. The patient had a fasting glucose of 143 mg/dL, Homeostasis Model Assessment of Insulin Resistance (HOMA-IR) of 5.2, and acylated hemoglobin (HbA1c) of 8%, consistent with T2DM. He was referred to endocrinology for management.

**Table 1:** Laboratory findings at presentation HOMA-IR: Homeostasis Model Assessment of Insulin Resistance, HbA1c: Acylated hemoglobin, LDL: Low-density lipoprotein, HDL: High-density lipoprotein.

Test	Result	Reference range	Interpretation
Fasting plasma glucose	143 mg/dL	70–99 mg/dL	Elevated
Fasting insulin (calculated)	≈14.7 µU/mL	2–10 µU/mL	Elevated
HOMA-IR	5.2	<2.0 (normal insulin sensitivity)	Elevated, insulin resistance
HbA1c	8.0 %	<5.7 % (normal); 5.7–6.4 % (prediabetes); ≥6.5 % (diabetes)	Elevated, consistent with diabetes
Total cholesterol	182 mg/dL	<200 mg/dL	Normal
LDL cholesterol	110 mg/dL	<130 mg/dL	Normal
HDL cholesterol	42 mg/dL	>40 mg/dL (men) / >50 mg/dL (women)	Borderline low
Triglycerides	168 mg/dL	<150 mg/dL	Mildly elevated
Serum creatinine	0.9 mg/dL	0.7–1.3 mg/dL	Normal
ALT (alanine aminotransferase)	32 U/L	<40 U/L	Normal

Given the disseminated presentation, a single lesional sample was obtained from scaling on the back for direct microscopy using methylene blue stain, which showed clusters of round spores and short hyphae, confirming infection by *Malassezia *spp. and the diagnosis of pityriasis versicolor (Figure 1C). Wood’s lamp and dermoscopy were not performed. 

Baseline liver function tests were within normal limits before therapy. Laboratory evaluation revealed a fasting plasma glucose of 143 mg/dL, HbA1c 8.0 %, HOMA-IR 5.2, and lipid profile as summarized in Table 1. The HOMA-IR index was calculated as:

\[ \text{HOMA-IR} = \frac{\text{Glucose (mg/dL)} \times \text{Insulin (µU/mL)}}{405} \]

Rearranging to estimate fasting insulin gives:

\[ \text{Insulin (µU/mL)} = \frac{\text{HOMA-IR} \times 405}{\text{Glucose (mg/dL)}} \]

Substituting the patient’s values (HOMA-IR 5.2, glucose 143 mg/dL) yields an estimated fasting insulin ≈ 14.7 µU/mL. For Latin American populations, values above 2.5 are widely accepted as indicative of insulin resistance, confirming marked metabolic impairment in this patient. Treatment consisted of oral itraconazole 100 mg every 12 hours for two weeks after confirming normal hepatic baseline values and absence of CYP3A4-interacting drugs. At the four-week follow-up, lesions showed complete clinical resolution without recurrence; repeat microscopy was not deemed necessary given full clearance. The patient was referred to endocrinology and initiated on metformin (850 mg twice daily) with dietary modification and weight-management counseling.

## Discussion

Relationship between pityriasis versicolor and type 2 diabetes mellitus

In this case, acanthosis nigricans prompted metabolic testing and revealed previously unrecognized T2DM (fasting glucose 143 mg/dL; HbA1c 8%; HOMA-IR 5.2). Although systemic factors such as insulin resistance may influence dissemination, local and environmental contributors are also relevant. The same factors that increase PV prevalence-heat, humidity, sweating, and occlusive clothing-could similarly facilitate fungal proliferation and spread in this patient. Acanthosis nigricans and the elevated HOMA-IR are therefore interpreted as contextual risk indicators for dissemination rather than evidence of a direct causal pathway.

The relationship between PV and T2DM remains controversial. Single-center studies have yielded conflicting findings: Thilak et al. (2017) reported PV in 17.1% of 400 Indian diabetics with superficial mycoses, ranking third after candidiasis and dermatophytosis [3]. In contrast, Joshua J (2017) found no excess DM among 200 PV cases [4], and Mansour et al. (2008) also reported no association in 300 diabetics versus controls [5].

More recent large-scale datasets provide a different perspective. A 2024 analysis of the All of Us database (32,679 PV cases) observed lower odds of PV among individuals with T2DM (odds ratio (OR) 0.68; 95% CI 0.50-0.93) [6], consistent with a 2025 commercial insurance database study showing OR 0.78 (95% CI 0.74-0.82) [7]. Taken together, these data suggest that T2DM is unlikely to be a primary predisposing factor for PV. Notably, odds ratios below 1 may reflect residual confounding or care-seeking patterns rather than true protection. Current data cannot determine whether, in cases such as our patient's, T2DM contributes to PV incidence or dissemination; therefore, we expand on mechanistic considerations below.

The odds ratios below 1 observed in large databases should be interpreted with caution, as they may reflect residual confounding, care-seeking or coding differences, or unmeasured over-the-counter antifungal use, and most estimates derive from prevalent rather than incident disease. While these epidemiologic findings suggest no positive association, they do not preclude coincidental co-occurrence with metabolic dysfunction, as seen in our patient. Causality cannot be inferred from a single case, but such presentations underscore the need for mechanistic exploration. Our observations are therefore hypothesis-generating, and we next outline a theoretical framework for how metabolic alterations might influence PV dissemination, recognizing that this requires prospective, biomarker-based validation.

Speculative working model: immune-metabolic and physicochemical elements

How might the associations between T2DM and PV diverge from those observed with dermatophytoses, given that both involve interleukin (IL)-17-dependent pathways? Dermatophyte infections rely predominantly on IL-17-mediated clearance, and T2DM is associated with broad innate and adaptive immune alterations that can increase susceptibility [8-10]. By contrast, *Malassezia*, a lipid-dependent commensal, depends on coordinated IL-23/IL-17 activity for effective containment. When this balance is suboptimal, fungal persistence may occur without overt inflammation [11,12]. In this speculative framework, T2DM-associated immune alterations may not uniformly predispose to PV. Instead, dissemination could reflect permissive interactions among epidermal lipid metabolism, immune tone, and physicochemical factors that together favor fungal proliferation despite otherwise competent immunity (Table 2).

**Table 2:** Hypothesized mechanistic distinctions between dermatophytoses and PV Dermatophytoses exemplify a direct “immune deficiency–infection” paradigm, in which impaired Th17 immunity increases susceptibility. By contrast, pityriasis versicolor (PV) appears to follow a “partial containment” model, wherein *Malassezia* overgrowth depends not only on immune tone but also on metabolic and physicochemical factors. In this speculative framework, disseminated PV may arise when permissive lipid environments and reduced inflammatory thresholds allow fungal proliferation despite otherwise intact overall immunity. Note: Right-column features represent a hypothesized framework derived from literature synthesis and author inference; these mechanisms remain to be empirically validated.

Feature	Dermatophytoses	PV – Proposed “Partial Containment” Model
Pathogen	Dermatophytes, obligate pathogens of keratinized tissue	*Malassezia *spp., lipid-dependent commensals of normal skin
Host–pathogen relationship	Exogenous infection; no commensal phase	Endogenous shift from commensal to pathogenic overgrowth
Primary immune control	Dominated by Th1/Th17 responses; IL-17 critical for clearance	IL-23/IL-17 axis contributes, but containment may also depend on lipid/barrier regulation and type-2 immune tone
Impact of T2DM and immune dysfunction	T2DM-associated immune alterations impair Th17 function, consistently increasing susceptibility and severity	Epidemiologic studies show no consistent association; dissemination may instead reflect altered lipid metabolism or immune tone rather than global susceptibility
Inflammatory profile	Marked: erythematous, scaly, annular plaques with prominent host response	Subtle: hypo- or hyperpigmented macules with minimal inflammation
Metabolic/lipid context	Limited role described; fungal growth largely driven by host immune status	PPARγ/LXR-driven lipid programs may increase free-fatty-acid availability; relative reductions in IL-13 and IL-17 activity could permit fungal overgrowth with minimal inflammation
Physicochemical contribution	Minimal role described	Lipid microdomains and hydrophobic clustering may passively raise local fungal density and complement immune–metabolic permissiveness
Paradigm	Direct relationship: impaired immunity predisposes to infection	Multifactorial regulation: commensalism, immune tolerance vs. clearance, metabolic shifts, and physicochemical aggregation jointly influence disease expression

Dermatophytoses exemplify a direct “immune deficiency-infection” paradigm, in which impaired Th17 immunity increases susceptibility. By contrast, PV appears to follow a “partial containment” model, wherein *Malassezia *overgrowth depends on immune modulation driven by metabolic reprogramming, a state that increases lipid availability while attenuating inflammatory signaling. In this view, disseminated PV may arise when lipid-rich microenvironments and low-inflammation conditions jointly allow fungal proliferation despite otherwise intact immune function. Within this context, epidermal lipid regulatory pathways (Peroxisome Proliferator-Activated Receptor gamma/Liver X Receptor (PPARγ/LXR)) may become relatively active, increasing free fatty acid availability while concurrently dampening IL-13 and IL-17-mediated clearance [13]. This combination could sustain high fungal density under minimal inflammation (“partial containment”).

**Figure 2:**
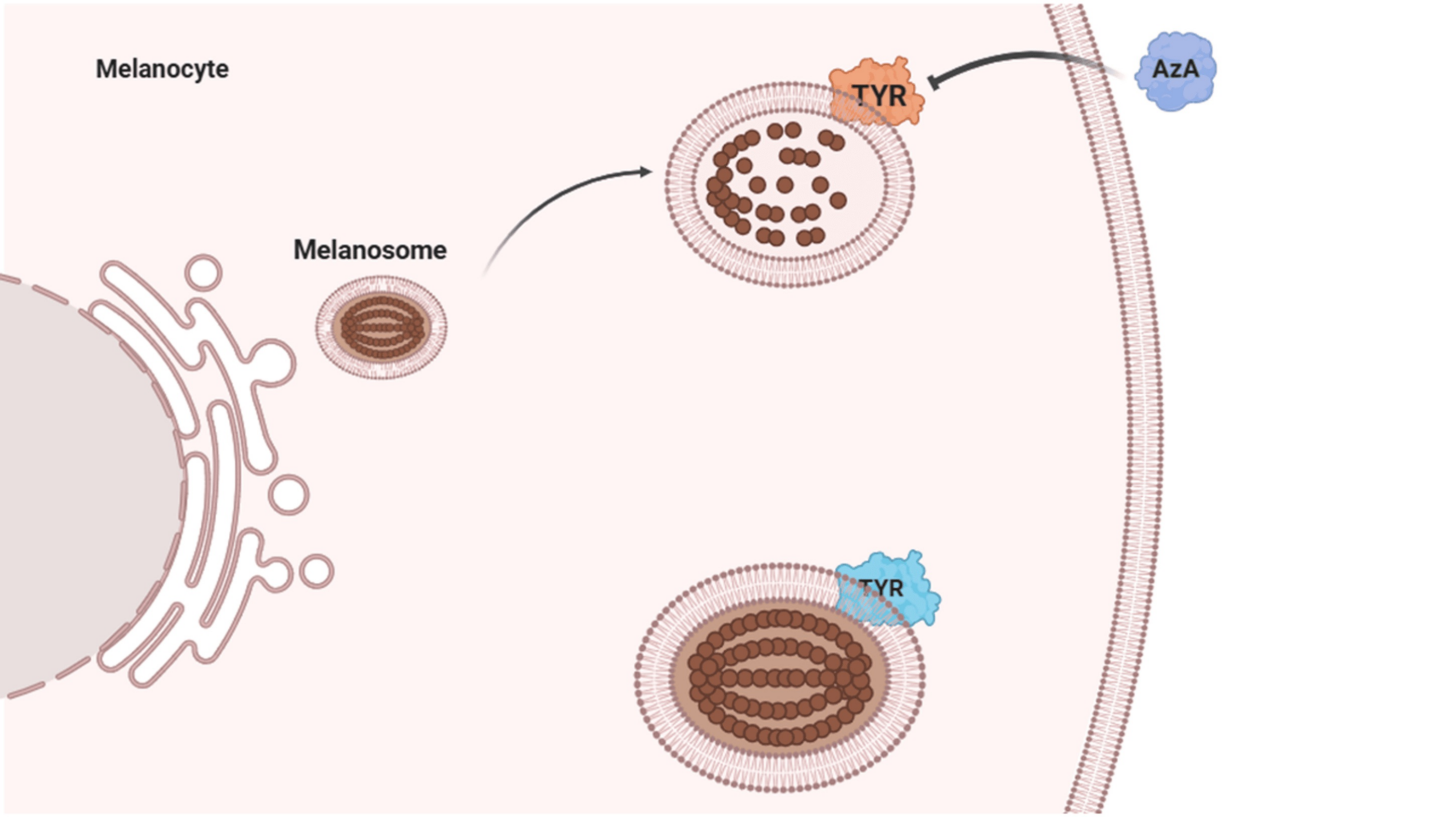
Mechanism of hypopigmentation in pityriasis versicolor *Malassezia *overgrowth in a lipid-rich, low-inflammation setting promotes production of azelaic acid (AzA). AzA inhibits tyrosinase (TYR) activity within melanosomes, suppressing melanogenesis and resulting in hypopigmented macules. This pigmentary alteration represents the final downstream effect of the “partial containment” model, in which permissive metabolic–immune conditions allow fungal proliferation despite limited host inflammation. Patient image published with written informed consent. Image created with BioRender.com.

From a physicochemical standpoint, lipid microdomains in the stratum corneum and around pilosebaceous units may reduce interfacial repulsion and promote hydrophobic aggregation, passively concentrating fungal cells [11,14]. In such lipid-rich, low-inflammation environments, *Malassezia *may reach densities sufficient to produce higher levels of dicarboxylic acids such as azelaic acid, which inhibit tyrosinase (TYR) within melanosomes, leading to hypopigmentation [1] (Figure 2). Collectively, these processes illustrate the “partial containment” model: a metabolically driven, low-inflammatory equilibrium that allows fungal proliferation and yields the characteristic pigmentary phenotype of pityriasis versicolor.

Clinical implications

For disseminated PV, given current evidence gaps regarding its relationship with metabolic disease, it is reasonable to screen for metabolic dysregulation (e.g., insulin resistance or T2DM) and to consider systemic therapy when topical regimens are impractical. Systemic treatment may be indicated in cases involving limb or widespread distribution, recurrent disease, or occupational factors such as heat, humidity, or occlusive clothing that limit topical efficacy.

Our patient achieved complete clinical resolution with oral itraconazole (100 mg twice daily for two weeks) alongside initiation of endocrine management, underscoring the value of multidisciplinary care in atypical or extensive presentations. Itraconazole should be used with standard precautions-avoiding drug interactions, assessing hepatic function before and during therapy, and exercising caution in patients with underlying cardiac disease.

Because PV relapses are common, monthly one-day itraconazole prophylaxis (200 mg twice on a single day each month for six months after initial cure) reduced recurrence rates versus placebo in a randomized, double-blind trial and can be considered for patients with frequent relapse or high exposure to humid, tropical environments [1,15]. Future studies should evaluate larger cohorts with mechanistic and biomarker-based approaches to clarify metabolic and immunologic determinants of dissemination.

Limitations and future directions

This report describes a single case without mechanistic or longitudinal biomarker measurements; causality between metabolic dysregulation and PV dissemination, therefore, cannot be established. Prospective, controlled studies with integrated lipidomic, cytokine, and mycological profiling are needed to validate the proposed immune-metabolic framework, clarify the role of metabolic dysfunction in fungal containment, and identify biomarkers predictive of dissemination.

## Conclusions

Disseminated PV is a rare clinical entity. Current evidence indicates that T2DM is not a consistent risk factor for PV; however, metabolic alterations associated with insulin resistance may promote dissemination through effects on epidermal lipid regulation and immune tone. As this report describes a single, hypothesis-generating case, causality cannot be established, and mechanistic assays or longitudinal metabolic follow-up were not performed. Additionally, environmental and local factors, including heat, humidity, sweating, and delayed consultation, may have contributed to disease spread. Within this speculative framework, we propose a “partial containment” model in which *Malassezia *proliferates in lipid-rich, low-inflammation conditions, producing dicarboxylic acids such as azelaic acid that inhibit tyrosinase and lead to hypopigmentation. Clinically, metabolic screening should be considered in disseminated PV, and systemic antifungal therapy may be warranted when topical options are impractical or relapse-prone, with standard precautions for itraconazole (drug interactions, hepatic monitoring, cardiac caution). Future prospective and biomarker-integrated studies are required to validate this immunometabolic model and define host determinants of dissemination.
